# Phenotypic Plasticity and Androgen Receptor Bypass Drive Cross-Resistance to Apalutamide in Castration-Resistant Prostate Cancer Cell Models

**DOI:** 10.3390/ijms26135939

**Published:** 2025-06-20

**Authors:** Iris Simon, Jose Manuel Sanchez-Manas, Sonia Perales, Gonzalo Martinez-Navajas, Jorge Ceron-Hernandez, Pedro J. Real

**Affiliations:** 1 Gene Regulation, Stem Cells and Development Group, GENyO, Pfizer-University of Granada-Andalusian Regional Government Centre for Genomics and Oncological Research, Avenida de la Ilustracion 114, PTS Granada, 18016 Granada, Spain; simonsaez.iris@mayo.edu (I.S.); jose.sanchez@genyo.es (J.M.S.-M.); sopero@ugr.es (S.P.); jorge.ceron@genyo.es (J.C.-H.); 2 Department of Biochemistry and Molecular Biology I, Faculty of Science, University of Granada, Avenida Fuentenueva s/n, 18071 Granada, Spain; 3 Instituto de Investigación Biosanitaria ibs.GRANADA, 18012 Granada, Spain; 4 Liquid Biopsies and Cancer Interception Group, GENyO, Pfizer-University of Granada-Andalusian Regional Government Centre for Genomics and Oncological Research, Avenida de la Ilustracion 114, PTS Granada, 18016 Granada, Spain

**Keywords:** prostate cancer, androgen deprivation therapy (ADT), castration-resistant prostate cancer (CRPC), Abiraterone, Enzalutamide, Apalutamide

## Abstract

The treatment of choice for prostate cancer is androgen deprivation (ADT) and novel hormonal agents such as Abiraterone, Enzalutamide, or Apalutamide. Initially, this therapy is highly effective, but a significant challenge arises as most patients eventually develop resistance, resulting in castration-resistant prostate cancer (CRPC). Furthermore, the sequential use of these drugs can lead to cross-resistance, diminishing their efficacy. Tumor heterogeneity plays a pivotal role in the development of resistance to different treatments. This study utilized cellular models of CRPC to assess the response to Apalutamide when it was administered as a second- or third-line treatment. Functional and genetic analyses were conducted in various CRPC cell models exposed to Apalutamide. These analyses included real-time cell monitoring assays, flow cytometry, clonogenicity assays, and RT-qPCR. CRPC cell models were capable of continued proliferation, maintained cell cycle profiles similar to those of untreated cells, and retained their clonogenic potential. Cross-resistance to Apalutamide in models of ADT, ADT plus Enzalutamide, or Abiraterone resistance did not correlate with the expression levels of *AR-V7* and *AR-V9* variants. Gene expression analysis of resistant prostate cancer cell lines revealed that treatment with Apalutamide induced the emergence of more aggressive phenotypes, including cancer stem cells or neuroendocrine differentiation profiles. Most CRPC cell models developed cross-resistance to Apalutamide and were able to proliferate and retain their clonogenic capability. Apalutamide resistance was not linked to the expression of *AR-V7* or *AR-V9* variants but was instead associated to bypass of AR signaling pathway and the emergence of more aggressive expression profiles.

## 1. Introduction

Prostate cancer (PCa) is the most frequently diagnosed tumor in nonsmoking men worldwide and the fifth leading cause of cancer-related death [1]. PCa progression is significantly influenced by androgen signaling through the androgen receptor (AR), which regulates the proliferation and growth of both healthy and cancerous prostate tissue [2].

Owing to this pivotal role, most current therapies are designed to target this pathway. In this context, the treatment of choice for PCa is androgen deprivation therapy (ADT) [3]. This therapy is initially very effective; however, most patients eventually develop resistance to this treatment, a condition known as castration-resistant prostate cancer (CRPC) [4,5]. These patients are subsequently treated with next-generation hormonal agents (NHAs), including Abiraterone acetate (AA), Enzalutamide (Enz) and Apalutamide (Apa) [3,6]. AA is an antiandrogen that inhibits testosterone synthesis via the inhibition of 17 α-hydroxylase (CYP17) [7]. In contrast, Enz and Apa are competitive inhibitors of the AR that act by binding to the Ligand Binding Domain of the AR and preventing its translocation and binding to the DNA necessary for AR activation [8,9]. However, these treatments also fail, as most patients have acquired common resistance mechanisms, and consequently, tumor progression continues. In addition, the use of these drugs sequentially generates cross-resistance, which results in a decrease in their effectiveness [7,8]. Therefore, the benefits of the use of combination therapies, rather than their sequential use, are currently being studied [10,11,12,13].

Different mechanisms of resistance to ADT have been described, and among them, the expression of variants of the androgen receptor (AR-Vs) is of particular importance [14]. One of the isoforms most frequently found in prostate tumors is *AR-V7* [15,16,17,18], which has recently been shown to be coexpressed with the *AR-V9* variant [19]. Likewise, tumor heterogeneity also plays a fundamental role in the acquisition of resistance to different treatments [20]. Increasing evidence suggests that prolonged inhibition of the AR pathway may alter the course of the disease, leading to histological dedifferentiation and alterations in the cell lineage in the form of tumor stem cells (CSCs), epithelial–mesenchymal transition (EMT), or neuroendocrine differentiation (NE) [21].

Knowing the molecular mechanisms involved in resistance to androgen deprivation therapies would allow us to predict the effectiveness of these new therapies and establish a new line of therapeutic action beneficial to PCa patients. Therefore, the main aim of this work was to evaluate the response to the use of Apa as second- or third-line treatment, using the basis of cellular models of PCa resistant to the most common androgen deprivation therapies. We evaluated the proliferation rates, cell cycle dynamics, AR isoforms expression, AR co-activators levels, AR target genes expression, clonogenic potential, and expression of phenotypic markers across the different CRPC models under Apa treatment.

## 2. Results

### 2.1. Proliferation Analysis of CRPC Models in Response to Apalutamide

**Figure 1:**
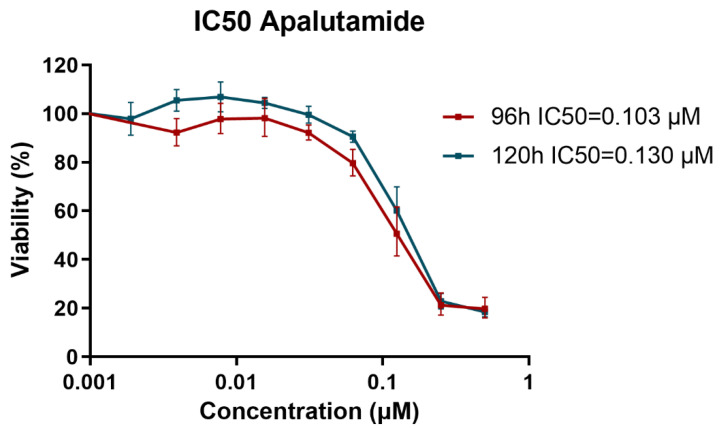
MTT assay for IC50 calculation. Determination of IC50 for between 4 and 5 days for Apa in the LNCaP WT cell line. The graph illustrates the percentage of viable cells across the range of drug concentrations tested. Data represent the mean ± SD calculated from quadruplicate samples for each drug concentration (n = 4; mean ± SD).

To establish a standard concentration of Apa for treating all resistant cell models, the IC50 values were determined using the most sensitive wild type (WT) cell line, LNCaP. An MTT assay was conducted from 3 to 5 days, testing a wide range of Apa concentrations spanning from 0.5 to 0.00097 µM (Figure 1). The IC50 for Apa was determined using GraphPad Prism^TM^ software, ranging from 0.103 µM at 4 days to 0.130 µM at 5 days. All subsequent experiments and analyses were conducted by treating all the cell lines with 0.130 µM Apa for 5 days.

**Figure 2:**
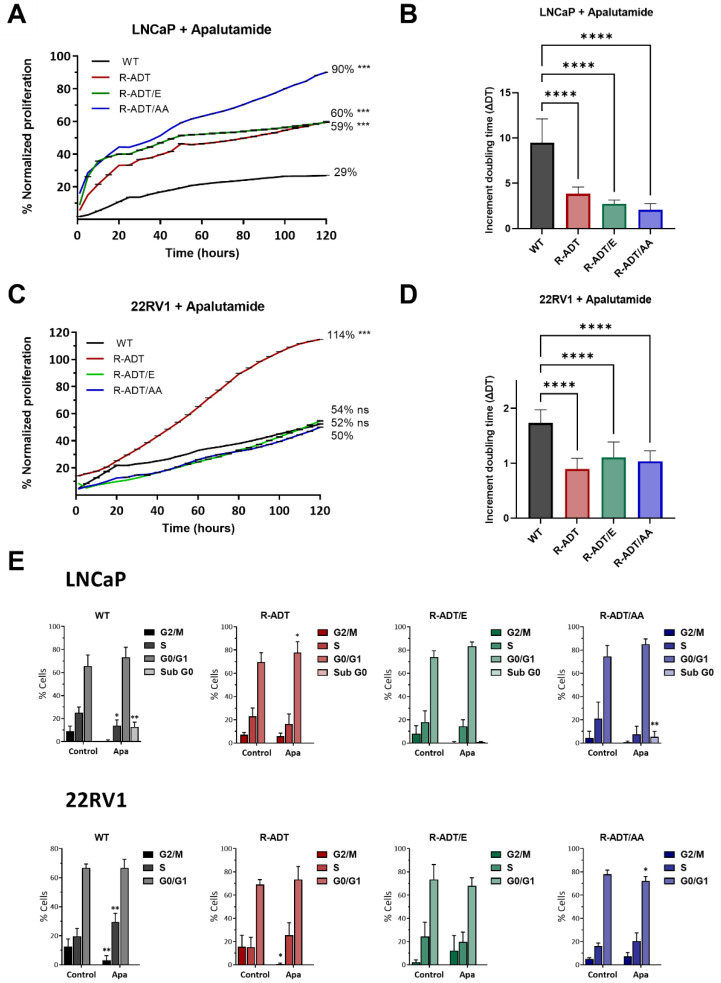
Analysis of the response of WT and CRPC cellular models to Apalutamide treatment. (**A**) Real-time cell proliferation analysis for LNCaP cell lines using the xCelligence system. The results have been standardized, with untreated control cell lines set as 100% (n = 4; mean ± SD). Statistics were assessed via Student’s *t* test (statistically significant differences: ns = non-significant, *** *p* < 0.001). (**B**) Cell doubling time. Data were normalized relative to untreated cell lines (n = 4; mean ± SD). Statistics were assessed via one-way ANOVA plus Dunnett’s multiple comparison test (statistically significant differences: **** *p* < 0.0001). (**C**) Real-time analysis of cell proliferation in 22RV1 cell lines using the xCelligence system. Data have been normalized, with untreated control cell lines designated as 100% (n = 4; mean ± SD). Statistics were assessed via Student’s *t* test (statistically significant differences: ns = non-significant, *** *p* < 0.001). (**D**) Cell doubling time. Data were normalized relative to untreated cell lines (n = 4; mean ± SD). Statistics were assessed via one-way ANOVA plus Dunnett’s multiple comparison test (statistically significant differences: **** *p* < 0.0001). (**E**) Cell cycle analysis with propidium iodide for WT and CRPC cellular models untreated or treated with 0.130 μM Apa. Bar charts represent the percentage of cells in different phases of the cell cycle (n = 6; mean ± SD). Statistics were assessed via two-way ANOVA plus Bonferroni’s multiple comparisons test (statistically significant differences: * *p* < 0.05, ** *p* < 0.01).

Next, the different cell lines were treated with 0.130 µM Apa for 5 days. When the effect of Apa exposure was studied, it was observed that, in the case of LNCaP cells, all the resistant cell lines (LNCaP R-ADT, LNCaP R-ADT/E, and LNCaP R-ADT/AA) presented greater proliferation rates than did the LNCaP WT line (*p* < 0.001). However, the sensitivity to such treatment was different in the different cell lines analyzed. In the LNCaP R-ADT line, Apa administration significantly reduced the cell proliferation rate (60% vs. 100%). Notably, in the LNCaP R-ADT/E line, a similar decrease in proliferation was also observed with respect to the untreated line (59% vs. 100%). Surprisingly, the proliferation rate of the LNCaP R-ADT/AA line treated with Apa barely decreased with respect to that of the same line without treatment (90% vs. 100%) (Figure 2A).

Additionally, analysis of the doubling times derived from these proliferation curves revealed a highly significant sensitivity of LNCaP WT cells to Apa treatment, with a substantial increase of 9.47 compared to untreated cells. In contrast, the impact of Apa on LNCaP CRPC models was more modest, with doubling time increases ranging from 3.83 in LNCaP R-ADT cells to 2.07 in LNCaP R-ADT/AA cells (Figure 2B).

The effects of Apa exposure on the resistance models of the 22RV1 cell line were also analyzed. The analysis of cell proliferation in the 22RV1 R-ADT cell line revealed the acquisition of cross-resistance to Apa, since the cells were able to proliferate normally in the presence of Apa (114% vs. 100%, +/− Apa). Furthermore, a highly significant increase in tolerance to Apa was observed compared to the 22RV1 WT cell line (114% vs. 50%) (*p* < 0.001). In contrast to what was observed in the LNCaP lines, the concomitant 22RV1 R-ADT/E and 22RV1 R-ADT/AA models presented a similar sensitivity to Apa as the 22RV1 WT cell line (54%, 52% vs. 50%) (Figure 2C).

In another analysis of cell proliferation, cell doubling time was evaluated in the different cell lines in the presence of Apa with respect to the untreated lines. In 22RV1 cell lines, the reduction in doubling time due to Apa treatment was less pronounced compared to LNCaP cell lines. Only the 22RV1 WT cells exhibited a significant delay, with a doubling time increase of 1.73 relative to untreated control. The CRPC cellular models largely maintained their proliferation rates, with values of 1.10 for 22RV1 R-ADT/E and 1.03 for 22RV1 R-ADT/AA cells, respectively. Notably, the 22RV1 R-ADT cell line displayed an unexpected response, increasing its proliferation rate under Apa exposure, as reflected by a reduced doubling time of 0.89 (Figure 2D).

Next, the 5-day cell cycle analysis revealed that LNCaP WT cells were initially sensitive to Apa treatment alone. Exposure to Apa induced arrest in the G_0_/G_1_ phase (*p* < 0.05); furthermore, this arrest was accompanied by the induction of cell death, as detected by the presence of a sub-G_0_ peak (*p* < 0.05). Surprisingly, the LNCaP R-ADT/AA line, which was tolerant of Apa at the proliferation level, presented cell cycle arrest at G_0_/G_1_ (*p* < 0.01) and slight induction of cell death (in the subG_0_ phase), as did the LNCaP WT line. In LNCaP R-ADT/E cells, there was no statistically significant reduction in the number of cells in G_2_/M, which was in accordance with the decrease in proliferation described above (Figure 2E). In contrast, LNCaP R-ADT cells presented a virtually unchanged cell cycle distribution in the presence or absence of Apa (Figure 2E).

With respect to cell cycle analysis, 22RV1 WT cells were sensitive to exclusive treatment with Apa. In this cell line, cell cycle arrest was observed in the G_0_/G_1_ phase (*p* < 0.01), which was not accompanied by cell death induction. Interestingly, 22RV1 R-ADT cells presented a reduction in the percentage of cells in G_2_/M, which was compensated by an increase in the percentage of cells in S phase. The remaining cell lines (22RV1 R-ADT/E and 22RV1 R-ADT/AA), which are cross-resistant, presented an unaltered cell cycle distribution in the presence of Apa (Figure 2E).

**Figure 3:**
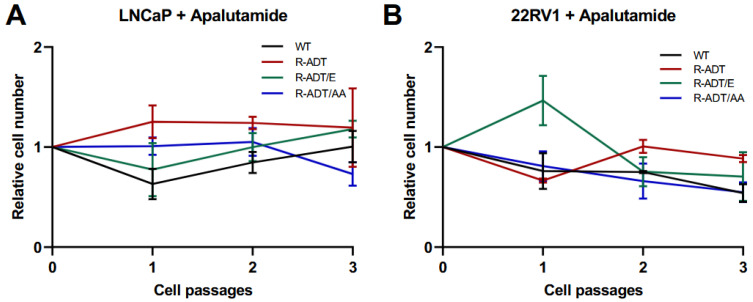
Sequential passage cell proliferation monitoring assay of WT and CRPC cellular models under apalutamide treatment. (**A**) Relative cell numbers after three consecutive passages in LNCaP WT and CRPC-resistant models treated with 0.130 µM Apa (n = 3; mean ± SD). (**B**) Corresponding analysis for 22RV1 WT and CRPC-resistant cell lines. Data were normalized to untreated control cell lines, which were set as 1 (n = 3; mean ± SD).

To determine the adhesion capability and the ability to resume proliferation under continuous Apa treatment, sequential passage cell proliferation monitoring assays were conducted during three consecutive passages. LNCaP WT cells, as observed at the proliferation level, initially showed sensitivity to Apa, but as the number of passages increased, their tolerance to treatment increased (0.6, 0.84 and 0.99) (Figure 3A). The behavior of the LNCaP resistance models varied across lines. In the LNCaP R-ADT line, tolerance to Apa remained stable across passages, consistently exceeding a normalized value of 1. Conversely, distinct responses were observed in the LNCaP R-ADT/E and LNCaP R-ADT/AA lines. In LNCaP R-ADT/E cells, the proliferation rate mirrored that of LNCaP WT cells, increasing from the second passage onward. In contrast, the LNCaP R-ADT/AA cells exhibited a progressive decrease in proliferation from the third passage onward, indicating an enhanced sensitivity to Apa with increasing passage number (Figure 3A).

On the other hand, in all 22RV1 cell lines that demonstrated some sensitivity to Apa (22RV1 WT, 22RV1 R-ADT/E, and 22RV1 R-ADT/AA), sensitivity consistently increased with successive passages. This effect was particularly pronounced in the 22RV1 R-ADT/E line, where relative proliferation compared to untreated cells decreased significantly across passages (1.44, 0.74 and 0.67, respectively) (Figure 3B). In contrast, the proliferation rates for the 22RV1 R-ADT line were comparable to those of the untreated line across all three passages (0.67, 1.00 and 0.89). These findings suggest that the 22RV1 R-ADT line maintains relative resistance to Apa, despite not having prior exposure to this NHA (Figure 3B).

Altogether, these results highlight the variable responses to Apa treatment across different PCa cell models and underscore the complex nature of antiandrogen resistance in CRPC.

### 2.2. Colony-Forming Capability Analysis of CRPC Models in Response to Apalutamide

**Figure 4:**
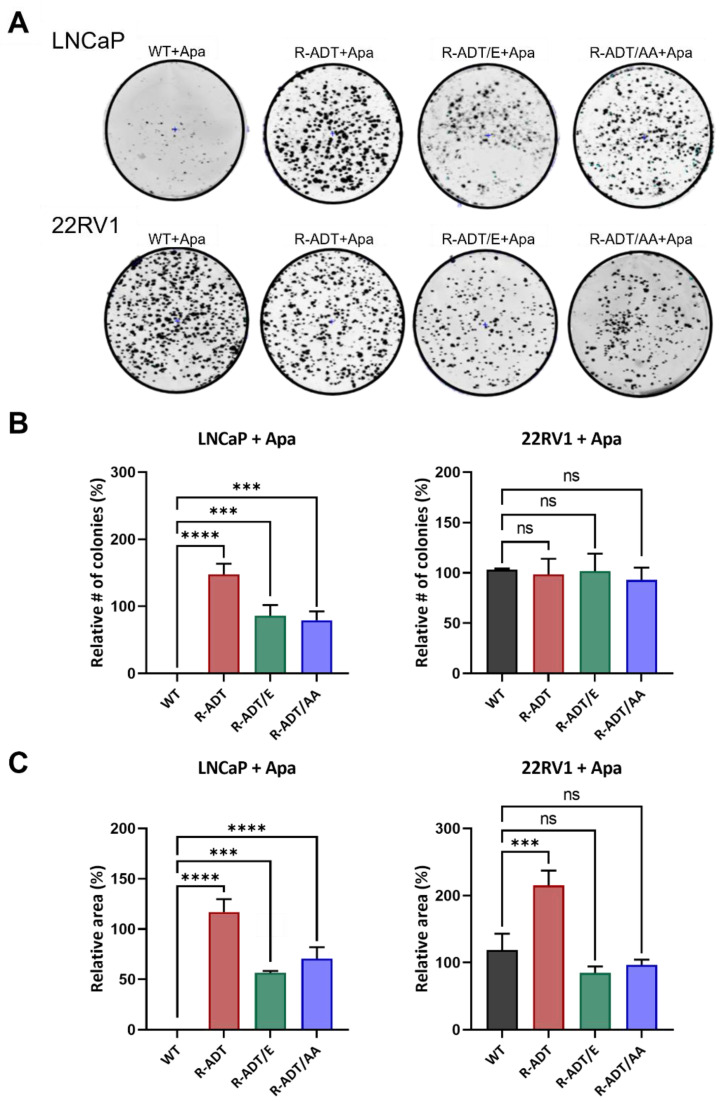
Clonogenic assays in PCa WT and CRPC cellular models treated with Apalutamide. (**A**) Representative images of colonies formed by the different cell lines under the specified conditions. (**B**) Colony count per well. Histogram data are normalized to untreated control cell lines (n = 3; mean ± SD). Statistics were assessed via one-way ANOVA plus Dunnett’s multiple comparison test (statistically significant differences: ns = non-significant, *** *p* < 0.001, **** *p* < 0.0001). (**C**) Colony area coverage in LNCaP and 22RV1 cellular models treated with 0.130 μM Apa. Histograms show the percentage of area occupied, normalized to the corresponding untreated control line (n = 3; mean ± SD). Statistics were assessed via one-way ANOVA plus Dunnett’s multiple comparison test (statistically significant differences: ns = non-significant, *** *p* < 0.001, **** *p* < 0.0001).

The colony-forming capability of the different cell lines in the presence of 0.130 μM Apa was analyzed in relation to that of the untreated lines, considering colonies from approximately 100 cells. The LNCaP WT line treated with Apa was unable to form colonies. In the case of the LNCaP R-ADT line, a tendency towards a greater number of colonies was observed, and the area of colony occupancy was even greater than that in the untreated cells. Compared with the untreated lines, the ADT resistance models and the second-line treatments (LNCaP R-ADT/E and LNCaP R-ADT/AA) treated with Apa presented slight reductions in both the number of colonies and area of occupation, with values of 56% (*p* < 0.001) for the LNCaP R-ADT/E line and 70% for the LNCaP R-ADT/AA line (Figure 4).

Similarly, the resistance patterns of the 22RV1 lines were analyzed. In the 22RV1 WT line treated with Apa, no differences were observed in either the number of colonies or the area of occupancy compared to the untreated line. The 22RV1 R-ADT cells in the presence of Apa presented a similar number of colonies to the untreated line; however, their area of occupancy was twice as large. The clonogenic capability of the 22RV1 R-ADT/E and 22RV1 R-ADT/AA lines in the presence of Apa did not significantly differ from that of the untreated lines in terms of either the number of colonies or the area of colony occupancy (Figure 4).

### 2.3. Gene Expression Analysis of AR Signaling Axis in CRPC Models in Response to Apalutamide

Next, we wanted to study the changes in gene expression that occurred in the different cell lines upon treatment with 0.130 μM Apa for 5 days.

**Figure 5:**
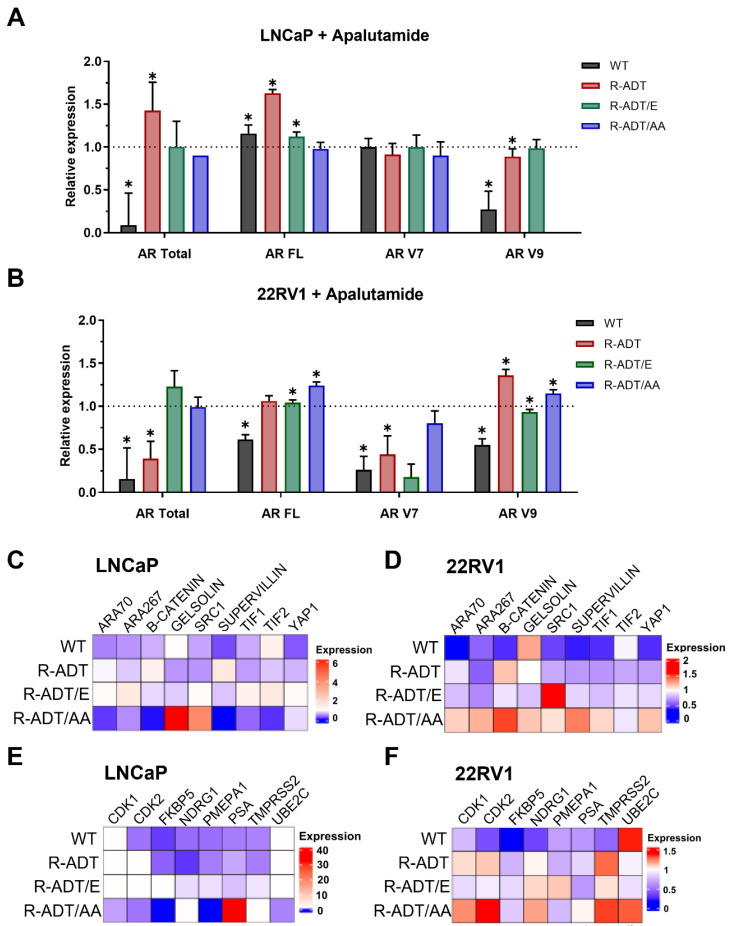
Quantification of *AR total*, *AR full length*, *AR-V7*, and *AR-V9* and AR coactivators and target genes in response to Apalutamide in CRPC cellular models. qPCR expression analysis for AR variants in LNCaP (**A**) and 22RV1 (**B**). Data were normalized to the endogenous control (GAPDH) and expressed relative to untreated cells (n = 3; mean ± SEM). Statistics were assessed via one-way ANOVA plus Dunnett’s multiple comparison test (statistically significant differences: * *p* < 0.05. Similarly, heatmap representation of qPCR expression analysis for AR Coactivators in LNCaP (**C**) and 22RV1 (**D**) or AR target genes in LNCaP (**E**) and 22RV1 (**F**). Data were normalized to the endogenous control (GAPDH) and expressed relative to untreated cells (n = 3), and the relative expression scales have been created for every PCa cell line.

First, we evaluated whether the presence of cross-resistance was related to the transcriptional reactivation of AR. To this end, we conducted a comprehensive analysis of the androgen signaling pathway and its coactivators (Figure 5 and Supplementary Table S2). We quantified the expression levels of key AR pathway components, including *AR FL*, *AR total*, and AR splice variants *AR-V7* and *AR-V9*. In addition, we evaluated a panel of AR coactivators (*Transforming Growth Factor Beta 1 Induced Transcript 1* (*ARA55*), *Nuclear Receptor Coactivator 4* (*ARA70*), *Nuclear Receptor Binding SET Domain Protein 1* (*ARA267*), β-*Catenin*, *Gelsolin*, *E1A Binding Protein P300* (*P300*), *Nuclear Receptor Coactivator 1* (*SRC1*), *Supervillin*, *Tripartite Motif Containing 24* (*TIF1*), *Nuclear Receptor Coactivator 2* (*TIF2*), and *Yes1 Associated Transcriptional Regulator* (*YAP1*)) to identify potential compensatory mechanisms or alternative pathways contributing to Apa resistance. Furthermore, we also analyzed the expression of androgen-responsive target genes (*Cyclin Dependent Kinase 1* (*CDK1*), *Cyclin Dependent Kinase 1* (*CDK2*), *FKBP Prolyl Isomerase 5* (*FKBP5*), *N-Myc Downstream Regulated 1* (*NDRG1*), *Prostate Transmembrane Protein, Androgen Induced 1* (*PMEPA1*), *Kallikrein Related Peptidase 3* (*PSA*), *Transmembrane Serine Protease 2* (*TMPRSS2*), and *Ubiquitin Conjugating Enzyme E2 C* (*UBE2C*)) to assess downstream signaling activity.

In LNCaP WT, which are sensitive to Apa, treatment significantly suppressed AR signaling. *AR total* and *AR-V9*, while *AR FL* and *AR-V7* were not modulated (Figure 5A). However, in 22RV1 WT, all the AR isoforms were downregulated after Apa treatment. In contrast, LNCaP R-ADT and 22RV1 R-ADT cells, resistant to ADT and Apa, showed distinct responses. In LNCaP R-ADT, *AR FL* and *AR total* expression increased significantly, suggesting compensatory AR axis activation. *AR-V7* levels remained unchanged, while *AR-V9* decreased slightly (Figure 5A). Conversely, in 22RV1 R-ADT cells, *AR total* remained suppressed, *AR-V7* decreased, but *AR-V9* was significantly elevated, indicating a potential role in cross-resistance (Figure 5B).

Among CRPC models, LNCaP R-ADT/E cells exhibited an intermediate molecular profile, with a moderate increase in *AR FL* and no significant changes in *AR total*, *AR-V7,* or *AR-V9*. In LNCaP R-ADT/AA cells, *AR FL*, *AR total*, and *AR-V7* remained unchanged; however, *AR-V9* was markedly reduced, suggesting partial resistance potentially driven by alternative regulatory mechanisms (Figure 5A). In 22RV1 R-ADT/E cells, both *AR-FL* and *AR total* levels were maintained or increased, indicating preserved AR signaling, while *AR-V7* was reduced. In 22RV1 R-ADT/AA cells, *AR-FL* and *AR-V9* were significantly upregulated, reflecting robust compensatory mechanisms sustaining Apa resistance (Figure 5B).

To further elucidate AR axis regulation, we analyzed coactivator expression. In WT cells, Apa induced broad downregulation of coactivators in both LNCaP and 22RV1 cell lines, except for *TIF1* upregulation in LNCaP WT and a notable induction of *P300* and *Gelsolin* in 22RV1 WT, potentially reflecting early compensatory responses (Figure 5C,D).

In LNCaP R-ADT cells, *β-Catenin* and *Supervillin* were upregulated, while *ARA267*, *P300*, *SRC1*, *TIF1*, *TIF2*, and *YAP1* were downregulated, consistent with partial AR axis reactivation (Figure 5C). Similarly, in 22RV1 R-ADT cells, most coactivators were downregulated, except for significant *β-Catenin* upregulation, suggesting a role in partial AR restoration (Figure 5D).

In the LNCaP R-ADT/E condition, the upregulation of *ARA70*, *ARA267*, *SRC1*, *TIF1*, *TIF2*, and *YAP1* indicated their potential role in sustaining AR transcription in the context of partial resistance (Figure 5C). In LNCaP R-ADT/AA cells, elevated *Gelsolin* and *SRC1*, alongside low levels of *ARA70, ARA267,* and *Supervillin*, supported the activation of a bypass pathway maintaining AR signaling despite the Apa blockade (Figure 5C). In 22RV1 R-ADT/E cells, *P300* and *SCR1* were upregulated, while other coactivators remained unchanged or slightly downregulated, suggesting their contribution of AR transcription in Apa resistance (Figure 5D). In contrast, 22RV1 R-ADT/AA cells displayed a markedly upregulation of *ARA70*, *ARA267*, *β-Catenin*, *Gelsolin*, and *Supervillin,* supporting a robust bypass strategy sustaining AR signaling despite Apa treatment (Figure 5D).

Finally, we assessed the expression of canonical AR target genes to evaluate downstream impact of AR axis modulation across the different cell models. In both LNCaP WT and 22RV1 WT cells, AR target genes, including *CDK1, CDK2, FKBP5, NDRG1, PMEPA1, PSA*, and *TMPRSS2*, were consistently downregulated, confirming effective AR axis inhibition (Figure 5E,F).

Conversely, in LNCaP R-ADT cells, proliferation-related genes (*CDK1, CDK2,* and *UBE2C*) were upregulated (Figure 5E), while in 22RV1 R-ADT cells, the upregulation of key cell cycle and signaling genes (*CDK1*, *CDK2* and *TMPRSS2*) suggested compensatory AR axis reactivation (Figure 5F).

In CRPC models, LNCaP R-ADT/E cells showed modest *CDK1* upregulation, with *MEPA1* and *TMPRSS2* repression (Figure 5E). In LNCaP R-ADT/AA cells, transcriptional repression of *CDK1*, *CDK2*, *FKBP5,* and *PMEPA1* contrasted with upregulation of *NDRG1*, TMPRSS2, and notably *PSA*, indicating a potent bypass mechanism with reprogrammed AR signaling (Figure 5E). In 22RV1 R-ADT/E cells, *PMEPA1* and *TMPRSS2* were significantly upregulated, while cell cycle progression and proliferation genes (*CDK1*, *CDK2*, *FKBP5*, and *UBE2C*) were repressed, reflecting heterogeneous responses and potential compensatory pathways despite partial suppression (Figure 5F). Lastly, in 22RV1 R-ADT/AA cells, proliferation and signaling-related genes (*CDK1*, *CDK2*, *TMPRSS2,* and *UBE2C)* were elevated, indicating robust AR signaling and resistance to Apa (Figure 5F).

Collectively, these findings demonstrate that Apa cross-resistance in PCa models is closely linked to tumor cells’ ability to maintain or reactivate androgen AR signaling through diverse molecular strategies.

### 2.4. Gene Expression Analysis of Different Cellular Phenotypes in CRPC Models in Response to Apalutamide

To investigate the combined effects of Apa exposure on CRPC models and control cell lines, we conducted a comprehensive gene expression analysis. This approach allowed us to assess how Apa influences the induction of different cellular phenotypes across these models. We evaluated markers associated with three key phenotypes using qPCR: CSC (*Nanog*, *POU Class 5 Homeobox 1* (*OCT3/4*), *SRY-Box Transcription Factor 2* (*SOX2*), *CD38* and *CD44*), EMT (*Vimentin*, *Integrin Subunit Alpha 2* (*ITGA2*), *SMAD Family Member 2* (*SMAD2*), *Snail Family Transcriptional Repressor* 1 (*SNAIL1*) and *E-Cadherin* (*CDH1*), and NE (*Chromogranin A* (*CHGA*), *Forkhead Box A2* (*FOXA2*), *Marker Of Proliferation Ki-67* (*Ki67*), *Neural Cell Adhesion Molecule 1* (*NCAM*), and *Enolase 2* (*NSE*)).

**Figure 6:**
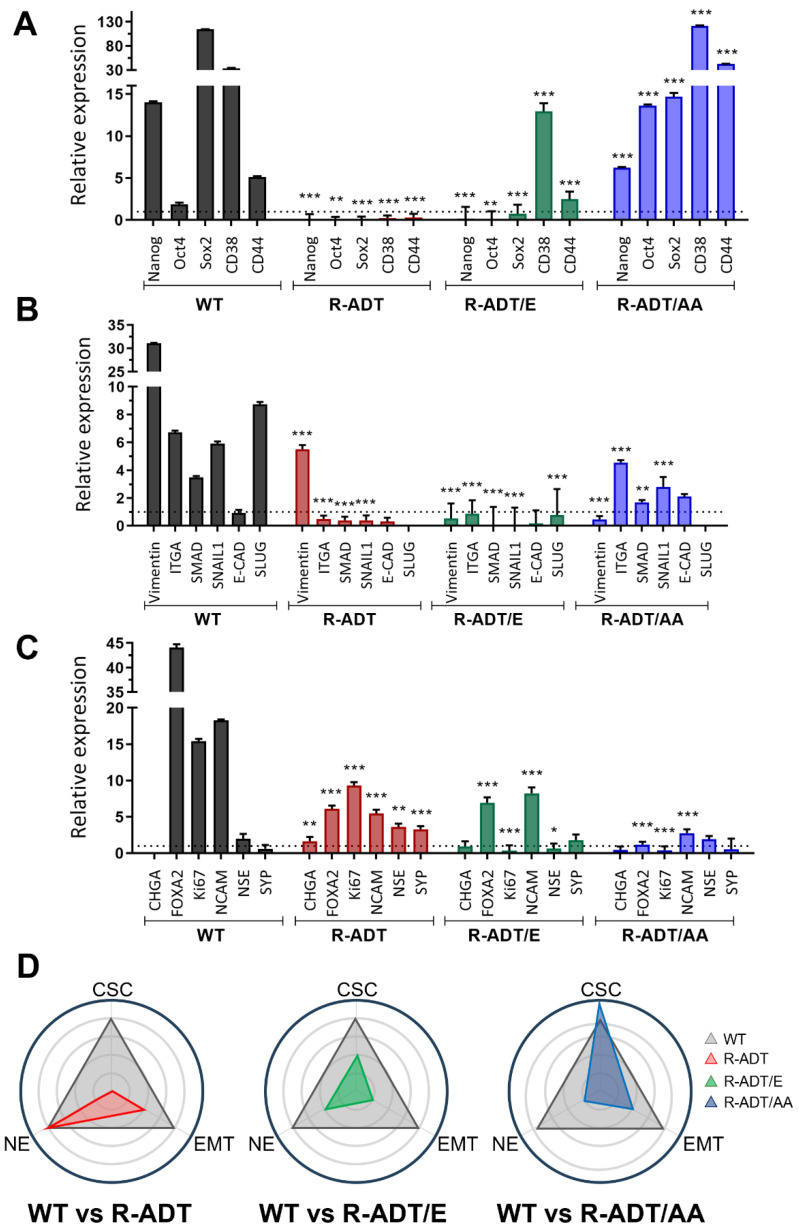
Quantification of differentiation markers associated with cancer stem cells (CSC), epithelial-to-mesenchymal transition (EMT), and neuroendocrine (NE) traits in LNCaP WT and CRPC cell lines treated with Apalutamide. qPCR expression analysis of CSC markers (**A**), EMT markers (**B**), and NE markers (**C**) in LNCaP WT, LNCaP R-ADT, LNCaP R-ADT/E, and LNCaP R-ADT/AA after 5 days of exposure to 0.130 μM Apa. Data were normalized to the endogenous control (GAPDH) and presented relative to untreated cells (n = 3; mean ± SEM). Statistics were assessed via two-way ANOVA plus Bonferroni’s multiple comparisons test (statistically significant differences; * *p* < 0.05, ** *p* < 0.01, *** *p* < 0.001). (**D**) Radial distribution plot illustrating the number of genes trending towards differentiation-specific profiles across LNCaP R-ADT, LNCaP R-ADT/E, and LNCaP R-ADT/AA cell lines compared to LNCaP WT cell line.

In LNCaP WT cells, 5-day Apa treatment led to a pronounced upregulation of four out of five CSC-related genes (*Nanog*, *SOX2*, *CD38,* and *CD44*) (Figure 6A). EMT markers also demonstrated significant changes, with *Vimentin* expression increasing 30-fold and *ITGA2*, *SMAD2,* and *SNAIL1* showing notable upregulation (Figure 6B). In contrast, *E-Cadherin* was slightly reduced, consistent with loss of epithelial characteristics. Additionally, NE phenotype markers, including *FOXA2*, *Ki67*, *NCAM,* and *NSE*, displayed increased expression levels (Figure 6C). To interpret and compare the tendency towards specific phenotypes across different cellular models, we created a plot illustrating the number of altered genes associated with each phenotype (Figure 6D). This visualization helps identify any skew towards particular phenotypic changes in response to Apa treatment. In LNCaP WT cells, the distribution of the three studied phenotypes, CSC, EMT, and NE, appeared to be uniformly balanced.

In contrast, LNCaP R-ADT cells exhibited a suppression of all CSC markers and four out of five EMT genes following 5-day exposure to Apa. However, a significant upregulation of NE markers, including *FOXA2*, *Ki67*, *NCAM,* and *NSE*, was observed (Figure 6A–C). The diagrammatic representation highlights a distinct skew towards the NE phenotype as a result of Apa treatment (Figure 6D, left panel).

The two LNCaP CRPC cell models exhibited markedly different expression profiles. LNCaP R-ADT/E showed an upregulation of two CSC markers (*CD38* and *CD44*) and two NE genes (*FOXA2* and *NCAM*) while repressing all EMT genes (Figure 6A–C). In contrast, LNCaP R-ADT/AA demonstrated a dramatic increase in all CSC markers (Figure 6A), a modest upregulation of two EMT genes (*ITGA2* and *SNAIL1*), and a slight increase in one NE marker (*NCAM*). Consequently, the LNCaP R-ADT/AA cell line displayed a pronounced shift towards a CSC phenotype (Figure 6D, right panel).

**Figure 7:**
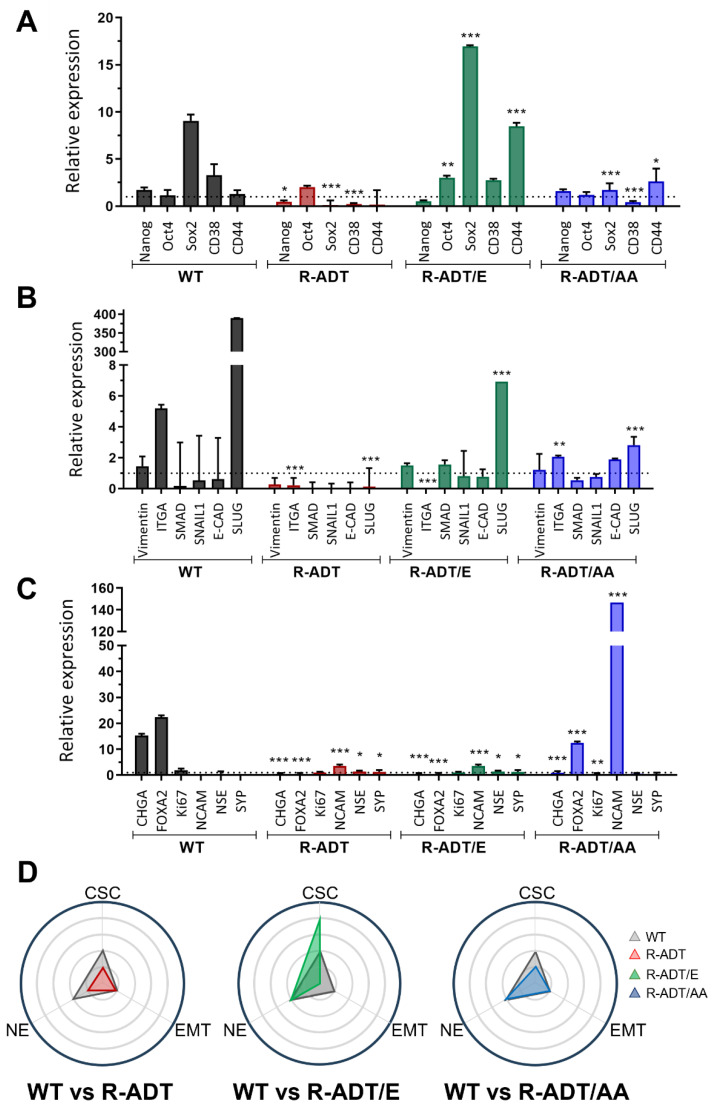
Expression analysis of differentiation markers for cancer stem cells (CSC), epithelial-to-mesenchymal transition (EMT), and neuroendocrine (NE) traits in 22RV1 WT and CRPC cell lines treated with Apalutamide. qPCR analysis of CSC markers (**A**), EMT markers (**B**), and NE markers (**C**) in 22RV1 WT, 22RV1 R-ADT, 22RV1 R-ADT/E, and 22RV1 R-ADT/AA cell lines after 5 days of treatment with 0.130 µM Apa. Data were normalized to the endogenous control (GAPDH) and expressed relative to untreated cells (n = 3; mean ± SEM). Statistics were assessed via two-way ANOVA plus Bonferroni’s multiple comparisons test (statistically significant differences: * *p* < 0.05, ** *p* < 0.01, *** *p* < 0.001). (**D**) Radial distribution plot showing the number of genes aligning with differentiation-specific trends in 22RV1 R-ADT, 22RV1 R-ADT/E, and 22RV1 R-ADT/AA cell lines compared to the 22RV1 WT line.

The gene expression profiles of 22RV1 cell lines showed distinct patterns compared to LNCaP cells when treated with 0.130 μM Apa for 5 days. 22RV1 WT cells induced the upregulation of two CSC markers (*SOX2* and *CD38*), one EMT marker (*ITGA2*), and two NE genes (*CHGA* and *FOXA2*) (Figure 7A–C). Consequently, this cell line exhibited a balanced distribution of the three different phenotypes, similarly to LNCaP WT cells (Figure 7D).

In contrast, most genes were downregulated in 22RV1 R-ADT cells, with only a slight upregulation of *OCT3/4* and *NCAM* (Figure 7A–C). Similarly, the 22RV1 R-ADT/AA cell line showed minimal changes, with *NCAM* as the only NE marker significantly upregulated (Figure 7A–C).

However, the 22RV1 R-ADT/E cell line demonstrated a dramatic shift towards a CSC phenotype, with four of five CSC markers (*OCT3/4*, *SOX2*, *CD38,* and *CD44*) upregulated, while all NE markers were downregulated (Figure 7A–C). As a result, there was a distinct skew towards the CSC phenotype following Apa exposure (Figure 7D, central panel).

Our findings highlight the diverse responses among all PCa cell lines, which can be attributed to their distinct resistance mechanisms and adaptations to androgen deprivation, either alone or in combination with NHAs.

## 3. Discussion

The development of NHAs has led to significant advances in the clinical management of CRPC, greatly improving survival rates for PCa patients. However, determining the optimal treatment sequence for CRPC remains challenging owing to the potential for cross-resistance between therapies. Cross-resistance, which is often a greater challenge than inherent resistance tied to individual genetic profiles, evolves in response to treatments and is influenced by tumor heterogeneity and clonal selection processes.

Cross-resistance typically arises following treatment with drugs that share similar mechanisms. For example, the sequential use of antiandrogens in CRPC may reduce the efficacy of these agents. Previous results from our group demonstrated that after the establishment of R-ADT/AA resistant PCa cellular model, sequential treatment with Enz did not reduce proliferation [22]. These findings indicated acquired cross-resistance between these NHAs, which has also been observed in previous studies on mCRPC patients [23,24,25]. Other studies have similarly shown that CRPC cells resistant to different NHAs exhibit cross-resistance to alternative treatments [26,27,28].

In clinical recommendations for CRPC management, Apa is approved for nonmetastatic CRPC following AA treatment and for the treatment of mCRPC [3,29,30]. The SPARTAN clinical trial further demonstrated that Apa improved metastasis-free survival in nonmetastatic CRPC patients [31]. Given the structural similarity of Apa to Enz, cross-resistance is expected to develop more readily following Enz exposure, with less impact following androgen deprivation therapy (ADT) or AA. While our resistant models generally exhibited increased Apa tolerance, the greatest tolerance was observed in the LNCaP R-ADT/AA and 22RV1 R-ADT cell lines, which maintained their proliferation and clonogenic capability in the presence of Apa. Consistent with our findings, Zhao et al. reported that resistance to Enz or AA in CRPC models also led to cross-resistance to Apa and vice versa [32]. These results suggest that combining NHAs may offer advantages over sequential use in CRPC management.

Indeed, a clinical study investigating the combination of AA and Apa in mCRPC demonstrated substantial antitumor activity [33]. Sustained exposure to second-line therapies in CRPC patients has been associated with resistance acquisition. Moreover, greater heterogeneity in circulating tumor cells (CTCs) correlates with poorer progression and overall survival in patients treated with antiandrogens. In line with these findings, we observed that Apa tolerance in LNCaP-resistant models increased with prolonged drug exposure. Interestingly, this effect was not detected in the 22RV1 lines; except for 22RV1 R-ADT cells, which presented cross-resistance to Apa, the other 22RV1 lines tended to display increased sensitivity to Apa over time. This underscores the high variability in treatment responses observed in clinical practice.

Additionally, our data provide further insights into the role of the AR in response to Apa. In sensitive cell lines, Apa effectively suppresses the AR signaling, as evidenced by the downregulation of AR isoforms and canonical target genes consistent with other AR-targeted therapies. However, Zhao et al. reported that Apa treatment did not alter AR expression in 22RV1 cells [32].

In resistant derivatives, distinct adaptive mechanisms emerge. LNCaP-resistant models, particularly under the R-ADT/AA condition, exhibit robust cross-resistance marked by PSA overexpression and coactivator reprogramming (notably *TIF2*, *Gelsolin*, and *SRC1*). Conversely, 22RV1-resistant models display a subtler resistance phenotype, with selective upregulation of *AR-V9* and coactivators such as *β-Catenin*, supporting a more stable yet less aggressive resistance profile. These differences underscore the variable dependence on *AR-FL*, AR splice variants, and AR transcriptional coregulators in mediating resistance. These results align with previous reports indicating that persistent AR signaling drives resistance to next-generation AR-targeted therapies, despite androgen suppression [34,35]. Importantly, the consistent involvement of *AR-V9*, *CDK1*, *CDK2*, and coactivators like *P300*, *ARA70*, *ARA267*, and *Supervillin* points to potential therapeutic targets. Furthermore, studies on AR coregulators have reinforced the idea of transcriptional reprogramming as a key player in resistance [36]. In line with this, Brooke et al. demonstrated that antiandrogens can act as selective AR modulators, inducing proteomic changes that do not necessarily correlate with gene expression levels [37]. Although our study did not assess protein expression, their findings support the idea that resistance may involve broader regulatory mechanisms beyond transcriptional control, further underscoring the complexity of the antiandrogen response.

These findings support our hypothesis that resistance to NHAs is linked to enhanced AR transcriptional activity. However, cross-resistance to additional treatments prevents further AR axis suppression. Taken together, our results suggest that monitoring AR isoform dynamics, coactivator expression, and AR target genes may provide critical insights for predicting treatment response and designing more effective combination strategies in CRPC.

However, resistance mechanisms in CRPC extend beyond just AR signaling alone. Increasing evidence suggests that the emergence of cross-resistance is not only driven by altered AR activity but also by broader transcriptional reprogramming, including pathways involved in EMT, CSC features, and NE differentiation [38]. These aggressive phenotypes contribute to the plasticity and heterogeneity of the disease, playing a critical role in the development of resistance to treatments. In line with these observations, recent studies have implicated transcriptional regulators like YAP in modulating these aggressive phenotypes, highlighting the complexity of cross-resistance mechanisms beyond direct AR signaling [39,40]. To explore these dynamics, we evaluated the expression profiles of these markers following treatment with Apa.

Interestingly, while both PCa WT cell lines treated with Apa exhibited a balanced distribution of the three phenotypic markers, resistance models displayed pronounced heterogeneity in their phenotypic profiles. This extensive variability and resistance to NHAs treatments observed in our models align with findings from numerous studies. Most notably, nearly all resistant cell lines demonstrated overexpression of CSC markers, supporting the hypothesis that CSCs are pivotal in initiating metastasis and facilitating transitions from epithelial to mesenchymal states [20,41]. Similarly, the presence of NE phenotype markers has been consistently identified in CRPC patients undergoing treatment with various NHAs [42,43].

One plausible explanation for this significant phenotypic diversity could lie in the clonal selection model. Substantial evidence has highlighted the adaptive advantage of clonal selection in androgen-independent cells [44]. For instance, after ADT, cells exhibiting CSC characteristics may gain a proliferative edge, allowing them to expand post-treatment [45]. Moreover, NE differentiation models have suggested that neuroendocrine progenitor cells (NEPCs) may arise from the differentiation of CSCs [45,46]. Similarly, the EMT process has been strongly associated with therapy resistance, metastasis formation, and increased disease aggressiveness [47]. Together, these findings underscore the complex interplay of cellular phenotypes in driving therapeutic resistance and disease progression in CRPC patients, suggesting that the presence of more aggressive phenotypes could contribute to resistance to different treatments.

## 4. Methods

### 4.1. Cell Culture

Two different human PCa cell lines obtained from the American Type Culture Collection (ATCC, Manassas, VA, USA) were used as controls: LNCaP (an androgen-sensitive adenocarcinoma cell line derived from supraclavicular lymph node metastasis) and 22RV1 (a cell line derived from an androgen-dependent CWR22 xenograft after castration-induced regression and relapse). Castration resistance cellular models were generated from these cell lines as previously described [22]. All the cell lines were maintained in RPMI 1640 medium (Sigma-Aldrich, Merck, St. Louis, MO, USA) supplemented with 1% Penicillin-Streptomycin Solution 100X (Biowest, Nuaille, France) and 10% fetal bovine serum (FBS)(Biowest) for the WT cell lines, while the castration-resistant cellular models used charcoal-stripped fetal bovine serum (CSS) in a humidified incubator at 37 °C with 5% CO_2_. Concomitant ADT-NHA-resistant PCa cell lines (R-ADT/E for ADT plus Enz, and R-ADT/AA for ADT plus AA) were grown in CSS-supplemented medium containing Enz (40 µM) or AA (20 µM), respectively. These concentrations were selected based on our previous study [22] and are consistent with concentrations reported in other studies [48,49,50,51,52]. All the cell lines were routinely tested for mycoplasma contamination using the Venor^®^GeM qEP (Minerva Biolabs, Dublin, Ireland) and authenticated via STR profiling using AmpFLSTR^®^ Identifiler^®^ Plus (Applied Biosystems, Thermo Fisher Scientific, Maltham, MA, USA), ensuring the presence of mycoplasma-free and STR-validated cells.

### 4.2. Treatment with Apalutamide as Second-Line Therapy

The IC50 value for Apa in LNCaP WT cells was determined via MTT assays (Sigma-Aldrich, Merck). Briefly, in 96-well plates, 1.5 × 10^4^ cells per well were seeded. After 24 h, the cells were treated with decreasing concentrations of Apa (0.5, 0.25, 0.125, 0.0625, 0.0312, 0.0156, 0.0078, 0.0039, 0.0019, and 0.00097 µM) for between 3 and 5 days. To measure cell viability, the medium was removed, and 100 µL of MTT reagent diluted in PBS was added to each well. After a 3 h of incubation, the resulting formazan crystals were dissolved by adding 100 µL of DMSO (Fisher Scientific, Waltham, MA, USA). The absorbance was then measured spectrophotometrically at 570 nm using a Tecan Infinite 200 Pro microplate reader (Tecan Trader AG, Männedorf, Switzerland). The IC_50_ values were calculated using GraphPad Prism^TM^ software (version 9.4.1 for Windows, San Diego, CA, USA). Cell viability data were first normalized to the untreated control (set as 100% viability). Non-linear regression analysis was then performed using the “log(inhibitor) vs. normalized response—Variable slope (four parameters)” model. The logarithm of the drug concentration was used to generate dose-response curves, from which IC_50_ values were automatically calculated by the software. WT and resistant cell lines were treated with the calculated IC50 for 5 days for proliferation, plating, cell cycle, colony formation assays, and gene expression analysis.

### 4.3. Cell Proliferation Assays

The effects of Apa on the proliferation and doubling time of both sensitive and resistant cell lines were evaluated via real-time cell monitoring assays (RTCA) using the xCELLigence system (ACEA Biosciences, Inc., San Diego, CA, USA). Cells were monitored over 5 days, and the impedance was recorded as a measure of the cell index (CI). At least four experimental replicates were performed for each experimental condition, and the results were analyzed in GraphPad Prism^TM^.

To calculate the doubling time, CI values over time were used to fit an exponential growth curve as indicated by the manufacturer. The doubling time was then determined using the standard equation:

$$Doubling\;time=\frac{\ln\left(2\right)}{slope\;of\;the\;exponential\;regression}$$

The slope was obtained by performing exponential fitting of the CI growth curve specifically during the logarithmic phase of cell growth. The normalized doubling time was calculated by dividing the doubling time of Apa-treated cells by the doubling time of their corresponding untreated controls for each cell line. This normalization process allows for a standardized comparison across all PCa cell lines, accounting for their inherent growth rates.

### 4.4. Sequential Passage Cell Proliferation Monitoring Assay

Initially, 1 × 10^5^ cells per well were seeded in 6-well plates and, 24 h later, the cells were treated with 0.130 µM Apa for 5 days. Following treatment, cells were detached, counted, and replated in the presence of Apa. This process was repeated over three consecutive passages. Each experimental condition was performed with a minimum of four replicates, and data were analyzed using GraphPad Prism^TM^.

### 4.5. Cell Cycle Experiments

Following Apa treatment, the cell cycle was assessed. The cells were dissociated after 5 days of culture, washed with PBS, and fixed in ice-cold 70% ethanol. After overnight incubation at −20 °C, cells were incubated with propidium iodide buffer (50 µg/mL propidium iodide, 200 µg/mL RNase, 0.05% NP-40, 3 mM EDTA pH 8.0 in PBS) and analyzed using a BD FACSVerse^TM^ flow cytometer (BD Biosciences, Franklin Lakes, NJ, USA). The fluorescence intensity representing ploidy (2N and 4N) was used to assess the G_0_/G_1_ and G_2_/M phases, respectively, using BDFACSDiva (version 9.0), ModFit LT^TM^ (version 6.0), and GraphPad Prism^TM^ software.

### 4.6. Expression Analysis of the AR Signaling Axis and Associated Transcriptional Coactivators

Total RNA was extracted post-Apa treatment via TRI Reagent (Life Technologies, Maltham, MA, USA). RNA concentration and purity were determined using a NanoDrop 2000c Spectrophotometer (Thermo Fisher Scientific), and reverse transcription was performed with 1 µg of total RNA via the Transcriptor First Strand cDNA Synthesis Kit (Thermo Fisher Scientific). qPCR analysis of the components of the androgen axis, including the AR (*AR Total*) and its variants Full Length (*AR FL*), *AR-V7*, and *AR-V9*, as well as a set of target genes and various AR coactivators, was conducted using iTaq Universal SYBR Green Supermix in an HT7900 Fast Real-Time PCR System (Applied Biosystems, Thermo Fisher Scientific) using custom primers (Supplementary Table S1). Relative expression (2^−ΔΔCt^) was calculated using *Glyceralhehide 3-phosphate deshidrogenase* (*GAPDH*) as a reference gene. The experiments were performed in triplicate (mean ± standard error mean (SEM)), and the results were statistically analyzed via Student’s *t* tests.

### 4.7. Phenotypic Marker Expression

qPCR analysis of CSC, EMT, and NE marker expression was performed following established methods and primers (Supplementary Table S1). Five genes for each phenotype were analyzed and plotted in sensitive and resistant cell lines. The experiments were performed in triplicate (mean ± SEM) as previously described.

### 4.8. Clonogenic Assays

Clonogenicity was assessed in 22RV1- and LNCaP-resistant models. The cells were seeded at 800 or 1000 cells/well, and after 24 h, cells were treated with Apa for 5 days and cultured for 12 days after treatment with fresh medium. Colonies were fixed in 70% ice-cold ethanol for 15 min and stained with 0.05% crystal violet solution. The wells were washed 3 times with water and allowed to dry completely. The clonogenic capability was quantified with an Odyssey infrared imaging system (LI-COR Biosciences; Lincoln, NE, USA) by measuring area and colony counts. Three experimental replicates were performed for each condition.

### 4.9. Statistical Analysis

The data are presented as the means ± standard deviation (SD) or standard error mean (SEM). Statistical comparisons were performed via unpaired Student’s *t* test and one-way Analysis of Variance (ANOVA) plus Dunnett’s multiple comparisons test or two-way ANOVA plus Bonferroni’s multiple comparisons test. All the statistical analyses and graph plotting were conducted via GraphPad Prism. Statistical significance was set at *p* < 0.05.

## 5. Conclusions

The varied responses observed across different cell lines to Apa treatment highlight the importance of understanding individual tumor characteristics. Each patient’s tumor may exhibit unique molecular features that significantly influence their response to treatment, underscoring the need for personalized approaches. The gene expression profiles and phenotypic changes identified in our cellular models hold predictive potential, offering valuable insights into how different patients may respond to therapy. These findings also emphasize the importance of considering a patient’s initial tumor profile, including genetic and phenotypic markers, when selecting the most suitable treatment strategy.

Moreover, the cellular models used in this study serve as effective tools for evaluating new drugs in development, shedding light on potential efficacy and resistance mechanisms. The diverse responses observed across various cell lines illustrate the many ways tumors can adapt to therapy, reinforcing the need for targeted treatment approaches. By integrating these insights into clinical practice, oncologists can make more informed decisions, potentially improving patient outcomes and minimizing the risk of ineffective therapies. This aligns with the principles of precision oncology, where treatments are tailored to the molecular characteristics of each patient’s tumor, paving the way for more personalized and effective cancer care.

## Data Availability

The data presented in this study are openly available in Zenodo at [10.5281/zenodo.14243094], reference number [14243094].

## Supplementary Materials

The following supporting information can be downloaded at: https://www.mdpi.com/article/10.3390/ijms26135939/s1.
