# Supplementary material for: Phenotypic Plasticity and Androgen Receptor Bypass Drive Cross-Resistance to Apalutamide in Castration-Resistant Prostate Cancer Cell Models
Source: Int J Mol Sci. 2025 Jun 20;26(13):5939. doi: 10.3390/ijms26135939 (PMC12250327; doi:10.3390/ijms26135939)
Supplement: Supplementary file 1 [file ijms-26-05939-s001.zip › ijms-3620752-supplementary.pdf]

Supplementary Table S1: Primer Sequences Used for qPCR Analysis

Gene Name	Fw (5′-3′)	Rv (5′-3′)
AR Total	CCACTTGTGTCAAAAGCGAA	AAGACCTGCCTGATCTGTGG
ARA267	CCGTGTAGGCCTTTTTGCAC	CCGTGGCAATGGGTTGATTC
ARA55	CCATCTAGCAAGGTGGCTTCA	ATGGTGTCTAGGCTGCCCTTTG
ARA70	GGGCAACCTCAGCCAGTTAT	CAAACTGCAGGGAGGCCATA
AR-Full Length	TGTCCATCTTGTCGTCTTCG	TTCAGATTACCAAGTTTCTTCAG
AR-V7	TGTCCATCTTGTCGTCTTCG	TAGTCTGGAGAAACCT
AR-V9	TGTCCATCTTGTCGTCTTCG	ACGTGATCCCAAAAGATGTG
β-Catenin	TCACTCCTCCTAATGGCTTG	GTTGCTGCCAGTGACTAACA
CD38	GCGATGCGTCAAGTACACTG	AGGTACGGTCTGAGTTCCCA
CD44	GGGAGTCAAGAAGGTGGAGC	AGCTCCATTGCCACTGTTGA
CDK1	TTTTCAGAGCTTTGGGCACT	CCATTTTGCCAGAAATTCGT
CDK2	CATTCCTCTTCCCCTCATCA	CAGGGACTCCAAAAGCTCTG
CHGA	GCTCCAAGACCTCGCTCTCC	CCTGATTGTTCCCCTCAGCCT
E-Cadherin	CCCGGGACAACGTTTATTAC	GCTGGCTCAAGTCAAAGTCC
FKBP5	TCCCTCGAATGCAACTCTCT	AAACATCCTTCCACCACAGC
FOXA2	CACCAACCCCACAAAATGGACC	CTCTCTCACTTGTCCTCGATCC
GAPDH	GAAGGTGAAGGTCGGAGT	GAAGATGGTGATGGGATTTC
Gelsolin	CAGTAGGCACCTTTCGTGGT	TTCCTCTCCTCCGTGTTTGC
ITGA	CCCGAGGGCATTGAAAACAC	CGGATAGTGCCCTGATGACG
Ki67	CTGGGTTACCTGGTCTTAGTTC	GAGGCTGTTCCTTGATGATTT
NANOG	TGCAGTTCCAGCCAAATTCTC	CTAGTGGTCTGCTGTATTACATTAAGG
NCAM	AGGAGACAGAAACGAAGCCA	GGTGTTGGAAATGCTCTGGT
NDRG1	ACAACCCTGAGATGGTGGAG	TGTGGACCACTTCCACGTTA
NSE	GGCACTCTACCAGGACTTTG	GCGATGACTCACCATAACCC
OCT3/4	AGTGAGAGGCAACCTGGAGA	ACACTCGGACCACATCCTTC
P300	CTGAGTGCTAACTGCGGGAC	GCGCGGGTTATGTAATGGTC
PMEPA1	AAGATGCCCTGTCCTCAGAA	GTGCTGCAGGTACGGATAGG
SMAD2	AGTGTGTAAAATTCCACCAG	ATTCTAGTTAGCTGATAGACGG
SNAIL1	GCTGCAGGACTCTAATCCAGA	ATCTCCGGAGGTGGGATG
SOX2	TCAGGAGTTGTCAAGGCAGAGAAG	CTCAGTCCTAGTCTTAAAGAGGCAGC
SRC-1	ACATCCCCAGCAACTACGTG	CAGTAGGCACCTTTCGTGGT
Supervillin	CCGCCAAGAGGTTGCTTTTC	CTGCAATGTGGCTGCGATG
TIF-1	GTGCCACCAGAACATCCAGA	AACTTGGGTGGCGAACGG

TIF-2	CAGCAAAGGGCAGACCAAAC	TGCTGTCCTGCAAGAGTCTG
TMPRSS2	CACTGTGCATCACCTTGACC	ACACGCCATCACACCAGTTA
UBE2C	ACCCAACATTGATAGTCCCTTG	GTAAAACGACGGCCAG
Vimentin	CCAGGCAAAGCAGGAGTC	CGAAGGTGACGAGCCATT
YAP1	TGACCCTCGTTTTGCCATGA	GTTGCTGCTGGTTGGAGTTG

Supplementary Table S2: qPCR Analysis for Heatmaps

**AR Co-activators**				
**Gene Name**	**Cell Line**	**Fold Induction**	**Cell Line**	**Fold Induction**
ARA70	LNCaP WT	0.50 ± 0.03 (p<0.05)	LNCaP R-ADT	0.96 ± 0.09
ARA267	LNCaP WT	0.58 ± 0.04 (p<0.05)	LNCaP R-ADT	0.79 ± 0.02 (p<0.05)
β-Catenin	LNCaP WT	0.68 ± 0.20 (p<0.05)	LNCaP R-ADT	1.35 ± 0.14 (p<0.05)
Gelsolin	LNCaP WT	1.06 ± 0.02 (p<0.05)	LNCaP R-ADT	0.58 ± 0.03 (p<0.05)
P300	LNCaP WT	0.60 ± 0.33 (p<0.05)	LNCaP R-ADT	0.29 ± 0.35 (p<0.05)
SRC-1	LNCaP WT	0.64 ± 0.12 (p<0.05)	LNCaP R-ADT	0.58 ± 0.08 (p<0.05)
Supervillin	LNCaP WT	0.28 ± 0.25 (p<0.05)	LNCaP R-ADT	1.62 ± 0.17 (p<0.05)
TIF-1	LNCaP WT	0.67 ± 0.09 (p<0.05)	LNCaP R-ADT	0.61 ± 0.05 (p<0.05)
TIF-2	LNCaP WT	1.40 ± 0.06 (p<0.05)	LNCaP R-ADT	0.77 ± 0.04 (p<0.05)
YAP1	LNCaP WT	0.34 ± 0.12 (p<0.05)	LNCaP R-ADT	0.70 ± 0.08 (p<0.05)
ARA70	LNCaP R-ADT/E	1.17 ± 0.02 (p<0.05)	LNCaP R-ADT/AA	0.21 ± 0.09 (p<0.05)
ARA267	LNCaP R-ADT/E	1.582 ± 0.02 (p<0.05)	LNCaP R-ADT/AA	0.54 ± 0.08 (p<0.05)
β-Catenin	LNCaP R-ADT/E	0.85 ± 0.20	LNCaP R-ADT/AA	0.10 ± 0.47 (p<0.05)
Gelsolin	LNCaP R-ADT/E	0.80 ± 0.20	LNCaP R-ADT/AA	6.21 ± 0.16 (p<0.05)
P300	LNCaP R-ADT/E	0.24 ± 0.23 (p<0.05)	LNCaP R-ADT/AA	0.83 ± 0.21
SRC-1	LNCaP R-ADT/E	1.10 ± 0.08 (p<0.05)	LNCaP R-ADT/AA	4.10 ± 0.11 (p<0.05)
Supervillin	LNCaP R-ADT/E	0.77 ± 0.12 (p<0.05)	LNCaP R-ADT/AA	0.08 ± 0.24 (p<0.05)
TIF-1	LNCaP R-ADT/E	1.36 ± 0.01 (p<0.05)	LNCaP R-ADT/AA	0.39 ± 0.16 (p<0.05)
TIF-2	LNCaP R-ADT/E	1.58 ± 0.03 (p<0.05)	LNCaP R-ADT/AA	0.19 ± 0.15 (p<0.05)
YAP1	LNCaP R-ADT/E	1.17 ± 0.04 (p<0.05)	LNCaP R-ADT/AA	0.85 ± 0.07 (p<0.05)
ARA70	22RV1 WT	0.35 ± 0.22 (p<0.05)	22RV1 R-ADT	0.92 ± 0.08
ARA267	22RV1 WT	0.56 ± 0.13 (p<0.05)	22RV1 R-ADT	0.55 ± 0.09 (p<0.05)
β-Catenin	22RV1 WT	0.42 ± 0.31 (p<0.05)	22RV1 R-ADT	1.18 ± 0.11 (p<0.05)
Gelsolin	22RV1 WT	1.26 ± 0.23 (p<0.05)	22RV1 R-ADT	1.00 ± 0.19
P300	22RV1 WT	5.98 ± 0.89 (p<0.05)	22RV1 R-ADT	0.54 ± 0.32 (p<0.05)

SRC-1	22RV1 WT	0.47 ± 0.23 (p<0.05)	22RV1 R-ADT	0.76 ± 0.09 (p<0.05)
Supervillin	22RV1 WT	0.38 ± 0.46	22RV1 R-ADT	0.71 ± 0.20 (p<0.05)
TIF-1	22RV1 WT	0.42 ± 0.23 (p<0.05)	22RV1 R-ADT	0.70 ± 0.07 (p<0.05)
TIF-2	22RV1 WT	0.97 ± 0.24	22RV1 R-ADT	0.75 ± 0.12 (p<0.05)
YAP1	22RV1 WT	0.42 ± 0.22 (p<0.05)	22RV1 R-ADT	0.73 ± 0.12 (p<0.05)
ARA70	22RV1 R-ADT/E	0.82 ± 0.07 (p<0.05)	22RV1 R-ADT/AA	1.14 ± 0.30 (p<0.05)
ARA267	22RV1 R-ADT/E	0.67 ± 0.36	22RV1 R-ADT/AA	1.23 ± 0.13 (p<0.05)
β-Catenin	22RV1 R-ADT/E	0.92 ± 0.14	22RV1 R-ADT/AA	1.50 ± 0.13 (p<0.05)
Gelsolin	22RV1 R-ADT/E	0.83 ± 0.30	22RV1 R-ADT/AA	1.17 ± 0.13 (p<0.05)
P300	22RV1 R-ADT/E	3.40 ± 1.10 (p<0.05)	22RV1 R-ADT/AA	0.72 ± 0.48
SRC-1	22RV1 R-ADT/E	1.57 ± 0.06 (p<0.05)	22RV1 R-ADT/AA	1.09 ± 0.13
Supervillin	22RV1 R-ADT/E	0.88 ± 0.10 (p<0.05)	22RV1 R-ADT/AA	1.37 ± 0.14 (p<0.05)
TIF-1	22RV1 R-ADT/E	0.82 ± 0.06 (p<0.05)	22RV1 R-ADT/AA	1.12 ± 0.12
TIF-2	22RV1 R-ADT/E	0.92 ± 0.08	22RV1 R-ADT/AA	0.93 ± 0.21
YAP1	22RV1 R-ADT/E	0.89 ± 0.11	22RV1 R-ADT/AA	1.18 ± 0.19
**AR Target Genes**				
**Gene Name**	**Cell Line**	**Fold Induction**	**Cell Line**	**Fold Induction**
CDK1	LNCaP WT	1,09 ± 0.09 (p<0.05)	LNCaP R-ADT	1,12 ± 0.04 (p<0.05)
CDK2	LNCaP WT	0,41 ± 0.11 (p<0.05)	LNCaP R-ADT	1,21 ± 0.07 (p<0.05)
FKBP5	LNCaP WT	0,20 ± 0.09 (p<0.05)	LNCaP R-ADT	0,34 ± 0.04 (p<0.05)
NDRG1	LNCaP WT	0,39 ± 0.09 (p<0.05)	LNCaP R-ADT	0,17 ± 0.06 (p<0.05)
PMEPA1	LNCaP WT	0,40 ± 0.11 (p<0.05)	LNCaP R-ADT	0,46 ± 0.04 (p<0.05)
PSA	LNCaP WT	0,45 ± 0.10 (p<0.05)	LNCaP R-ADT	0,64 ± 0.04 (p<0.05)
TMPRSS2	LNCaP WT	0,48 ± 0.10 (p<0.05)	LNCaP R-ADT	0,46 ± 0.04 (p<0.05)
UBE2C	LNCaP WT	1,13 ± 0.10 (p<0.05)	LNCaP R-ADT	1,39 ± 0.05 (p<0.05)
CDK1	LNCaP R-ADT/E	1,08 ± 0.04 (p<0.05)	LNCaP R-ADT/AA	0,61 ± 0.07 (p<0.05)
CDK2	LNCaP R-ADT/E	1,00 ± 0.14	LNCaP R-ADT/AA	0,43 ± 0.10 (p<0.05)
FKBP5	LNCaP R-ADT/E	1,23 ± 0.05 (p<0.05)	LNCaP R-ADT/AA	0,04 ± 0.10 (p<0.05)
NDRG1	LNCaP R-ADT/E	0,86 ± 0.04 (p<0.05)	LNCaP R-ADT/AA	1,42 ± 0.13 (p<0.05)
PMEPA1	LNCaP R-ADT/E	0,87 ± 0.07 (p<0.05)	LNCaP R-ADT/AA	0,04 ± 0.21 (p<0.05)
PSA	LNCaP R-ADT/E	0,74 ± 0.43	LNCaP R-ADT/AA	32,01 ± 0.07 (p<0.05)
TMPRSS2	LNCaP R-ADT/E	0,89 ± 0.08 (p<0.05)	LNCaP R-ADT/AA	1,51 ± 0.10 (p<0.05)
UBE2C	LNCaP R-ADT/E	1,04 ± 0.05	LNCaP R-ADT/AA	0,48 ± 0.20 (p<0.05)
CDK1	22RV1 WT	0,76 ± 0.06 (p<0.05)	22RV1 R-ADT	1,07 ± 0.06 (p<0.05)
CDK2	22RV1 WT	0,38 ± 0.08 (p<0.05)	22RV1 R-ADT	1,11 ± 0.06 (p<0.05)
FKBP5	22RV1 WT	0,18 ± 0.06 (p<0.05)	22RV1 R-ADT	0,73 ± 0.07 (p<0.05)
NDRG1	22RV1 WT	0,34 ± 0.07 (p<0.05)	22RV1 R-ADT	1,02 ± 0.06

PMEPA1	22RV1 WT	0,66 ± 0.06 (p<0.05)	22RV1 R-ADT	0,71 ± 0.06 (p<0.05)
PSA	22RV1 WT	0,63 ± 0.09 (p<0.05)	22RV1 R-ADT	0,85 ± 0.06 (p<0.05)
TMPRSS2	22RV1 WT	0,46 ± 0.07 (p<0.05)	22RV1 R-ADT	1,28 ± 0.11 (p<0.05)
UBE2C	22RV1 WT	1,37 ± 0.10 (p<0.05)	22RV1 R-ADT	0,95 ± 0.07
CDK1	22RV1 R-ADT/E	0,87 ± 0.03 (p<0.05)	22RV1 R-ADT/AA	1,22 ± 0.05 (p<0.05)
CDK2	22RV1 R-ADT/E	0,93 ± 0.05 (p<0.05)	22RV1 R-ADT/AA	1,38 ± 0.03 (p<0.05)
FKBP5	22RV1 R-ADT/E	0,86 ± 0.04 (p<0.05)	22RV1 R-ADT/AA	0,81 ± 0.19 (p<0.05)
NDRG1	22RV1 R-ADT/E	1,08 ± 0.09	22RV1 R-ADT/AA	1,18 ± 0.04 (p<0.05)
PMEPA1	22RV1 R-ADT/E	1,11 ± 0.05 (p<0.05)	22RV1 R-ADT/AA	0,76 ± 0.03 (p<0.05)
PSA	22RV1 R-ADT/E	0,64 ± 0.51	22RV1 R-ADT/AA	1,02 ± 0.07
TMPRSS2	22RV1 R-ADT/E	1,06 ± 0.03 (p<0.05)	22RV1 R-ADT/AA	1,34 ± 0.09 (p<0.05)
UBE2C	22RV1 R-ADT/E	0,89 ± 0.05 (p<0.05)	22RV1 R-ADT/AA	1,30 ± 0.03 (p<0.05)
